# The importance of saturating density dependence for population-level predictions of SARS-CoV-2 resurgence compared with density-independent or linearly density-dependent models, England, 23 March to 31 July 2020

**DOI:** 10.2807/1560-7917.ES.2021.26.49.2001809

**Published:** 2021-12-09

**Authors:** Emily S Nightingale, Oliver J Brady, CMMID Covid-19 working group, Laith Yakob, CMMID Covid-19 working group, Amy Gimma, Mark Jit, Christopher I Jarvis, Naomi R Waterlow, Simon R Procter, Megan Auzenbergs, Damien C Tully, David Simons, Akira Endo, Joel Hellewell, Rachel Lowe, Anna M Foss, Kevin van Zandvoort, Carl AB Pearson, Alicia Showering, Petra Klepac, Graham Medley, Billy J Quilty, Charlie Diamond, W John Edmunds, Alicia Rosello, Rosanna C Barnard, Kaja Abbas, Katharine Sherratt, Jack Williams, Sophie R Meakin, Matthew Quaife, Timothy W Russell, C Julian Villabona-Arenas, Kiesha Prem, Fiona Yueqian Sun, Nicholas G Davies, Rosalind M Eggo, Gwenan M Knight, Adam J Kucharski, Frank G Sandmann, Sebastian Funk, Georgia R Gore-Langton, Stefan Flasche, Thibaut Jombart, Hamish P Gibbs, Yang Liu, Oliver Brady, Nikos I Bosse, Yung-Wai Desmond Chan, Sam Abbott, Samuel Clifford, Katherine E Atkins, Emily S Nightingale, James D Munday

**Affiliations:** 1 Department of Global Health and Development, Faculty of Public Health and Policy, London School of Hygiene & Tropical Medicine, London, United Kingdom; 2 Centre of Mathematical Modelling for Infectious Diseases, London School of Hygiene & Tropical Medicine, London, United Kingdom; 3 The members of the group are listed under Investigators; 4 Department of Disease Control, Faculty of Infectious & Tropical Medicine, London School of Hygiene & Tropical Medicine, London, United Kingdom

**Keywords:** mathematical model, population Density, COVID-19

## Abstract

**Background:**

Population-level mathematical models of outbreaks typically assume that disease transmission is not impacted by population density (‘frequency-dependent’) or that it increases linearly with density (‘density-dependent’).

**Aim:**

We sought evidence for the role of population density in SARS-CoV-2 transmission.

**Methods:**

Using COVID-19-associated mortality data from England, we fitted multiple functional forms linking density with transmission. We projected forwards beyond lockdown to ascertain the consequences of different functional forms on infection resurgence.

**Results:**

COVID-19-associated mortality data from England show evidence of increasing with population density until a saturating level, after adjusting for local age distribution, deprivation, proportion of ethnic minority population and proportion of key workers among the working population. Projections from a mathematical model that accounts for this observation deviate markedly from the current status quo for SARS-CoV-2 models which either assume linearity between density and transmission (30% of models) or no relationship at all (70%). Respectively, these classical model structures over- and underestimate the delay in infection resurgence following the release of lockdown.

**Conclusion:**

Identifying saturation points for given populations and including transmission terms that account for this feature will improve model accuracy and utility for the current and future pandemics.

## Introduction

Like many pathogens that cause respiratory diseases [1-3], severe acute respiratory syndrome coronavirus 2 (SARS-CoV-2) appears to be transmitted more effectively in densely populated areas [4-6]. The increased disease rates reported among high-density populations [4,5,7,8] may, however, be an artefact of confounders, such as a higher proportion of individuals of lower socioeconomic status or from minority ethnic groups in urban areas [9]. Using coronavirus disease (COVID-19)-associated mortality data from the Office for National Statistics, we aimed to assess the evidence for density dependence.

Standard transmission models that either do or do not account for this density dependence have been used interchangeably because their projections are generally equivalent when population density remains unperturbed or is homogeneous, e.g. at a national level. While the ca 1% infection fatality rate for COVID-19 [10] is insufficient to destabilise populations, the reaction of most countries’ governments to curtail disease spread through lockdown and physical distancing has had unprecedented impacts on the density of mobile human populations. For example, the United Kingdom’s lockdown, which came into effect on 23 March 2020, effectively reduced the freely moving population from 66.5 million to 10.6 million (key workers) [11]. This same intervention was employed by numerous countries, similarly impacting their mobile populations [12]. We evaluate the extent to which models built to inform the epidemiology of COVID-19 use an underlying structure that can accommodate the drastic changes and variation in densities experienced by most global populations.

As lockdowns were gradually released over the latter part of 2020, global populations were expected to re-equilibrate to a ‘new normal’ whereby densities of mobile people were increased but in which contact patterns were expected to remain reduced through physical distancing interventions [13]. Using a suite of mathematical models, we illustrate the impact that the different, routinely ignored, assumptions underlying transmission and density may have in projecting infection dynamics and measuring intervention effectiveness.

## Methods

### Data

Reported COVID-19-related deaths between 1 March and 31 July 2020 were obtained in anonymised linelist form from Public Health England and were filtered to include all deaths which occurred within 28 days of positive COVID-19 test (n = 36,311). We aggregated individual records to lower-tier local authority (LTLA), and nationally by 10-year age bands in order to calculate age-standardised expected counts.

Local authority shapefiles and single-age population estimates were obtained from the Office for National Statistics [14]. Four sub-regions of Buckinghamshire (Aylesbury Vale, Chiltern, South Bucks, Wycombe) were aggregated in order to match most recent population estimates. The City of London was aggregated with Westminster because of its very small resident population, and the Isles of Scilly were excluded since no COVID-19-related deaths had been reported there during the study period. Index of multiple deprivation (IMD) [15], percentage of minority ethnic population [16] and percentage of key workers among the working population [17] are characteristics of the LTLA population potentially associated with both COVID-19 mortality and population density, therefore we included them as covariates in all models. Percentage of key workers was missing for Westminster and Cornwall; these were imputed by the median value across all neighbouring LTLA.

### Statistical analysis

Negative binomial regression models were fitted to the number of deaths (*n*) per LTLA, adjusting initially for the three covariates (IMD, % minority population, % key workers) and subsequently adding a fourth covariate, namely the lag in weeks behind the first death nationally. We adjusted for age distribution within the LTLA via inclusion of age-adjusted expected deaths (*E*) as an offset; these were calculated according to national age-specific rates (deaths per 100,000 per age band) applied to local population estimates in 10-year age bands. We accounted for population density in one of four functional forms: (i) constant/independent of population density, (ii) linear, (iii) log-linear and (iv) saturating.

For observed number of deaths (*D*), age-adjusted expected deaths (*E*) and defining *x_i_^IMD^*, *x_i_^mino^, x_i_^KW^, x_i_^lag^* and* x_i_^dens^*, respectively, as the deprivation score, % minority population, % key workers, lag in weeks behind first death nationally and population density of LTLA this yields the following model specification:


$$D_{i}\;\sim NegBin(\mu_{i}E_{i},\phi )$$

$$log(\mu_{i})=\beta_{0}+\beta_{1}x_{i}^{IMD}+\beta_{2}x_{i}^{mino}+\beta_{3}x_{i}^{KW}+\beta_{4}x_{i}^{lag}+\beta_{5}f_{k}(x_{i}^{dens})$$

where *μ_i_* is the rate relative to the expected, *ϕ* the size parameter (1/overdispersion) of the negative binomial distribution and *β_j_* the regression coefficients. The functional form of population density is defined as


$$f_{k}(x)=\left\{\begin{array}{c} 0,\;k=A\\ x,\;k=B\\ \log\left(x\right),\;k=C\\ sat\left(x,\;\theta \right),\;k=D \end{array}\right.$$

where


$$sat\left(x,\;\theta \right)=\frac{2\theta x}{1+2\theta x+\sqrt{(1+4\theta x})\;}$$

Models were fitted using the *rstanarm* package [18] with default weakly-informative priors. We compared the four model variants on leave-one-out information criterion (LOOIC), calculated via approximate leave-one-out cross-validation as implemented in the *loo* package [19]. Interpretation was the same as that of the Akaike information criterion in that smaller values reflected better fit. The value of *ϴ* for the saturating function was determined by manual optimisation of the generalised linear model with respect to LOOIC on a hold-out set of 40% of LTLA, over a range from 0.001 to 1.

For the saturating model, the impact of an 84% reduction in effective population density as a result of lockdown on predicted mortality rates among the freely moving population was calculated as a percentage change between mean model-predicted deaths under the original and reduced densities.

### Mathematical model

**Figure 1:**
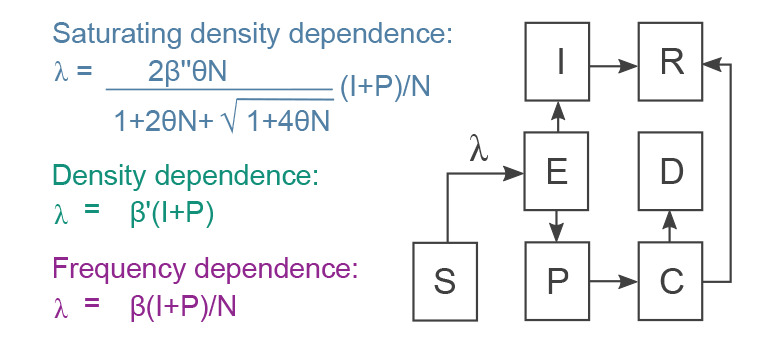
SARS-CoV-2 transmission model compartments and alternative transmission assumptions, England, March–July 2020

We use a discrete-time, deterministic compartmental model (Figure 1) with daily timesteps to simulate SARS-CoV-2 transmission. From the first day of lockdown (23 March 2020), we assumed 84% of the population to enter isolation in which frequency-dependent transmission occurs. We made this assumption for the lockdown sub-population because an individual’s likelihood of contracting infection while in their home is limited by their household size (i.e. not impacted by the density of individuals under isolation in different households). Each model was fitted independently to England’s COVID-19-associated mortality data (up until 1 August 2020). We compared frequency-dependent and both linearly and saturating density-dependent transmission for when lockdown is released. We also explored the impact of varying rates of connectivity between locked-down and free-moving individuals because those under lockdown were still afforded some freedom of movement, and because key workers potentially cohabit with those under lockdown (Supplementary Figure S1 shows limited impact of doubling the rate of this connectivity). We compared frequency-dependent and linearly density-dependent transmission (the limiting cases for the saturating density-dependent model [20]) among the remaining free-movers for a range of lockdown release schedules (over a period of between 1 and 12 months). Contact rates are reduced through two distinct mechanisms under the density-dependent models: whereas reduced contact through physical distancing and behavioural changes among the freely moving population (e.g. the 2 m rule) was included in all models, only the density-dependent versions assumed reduced opportunities for mobile people coming into contact with other mobile people because of their substantially depleted numbers. Full model specification and sources for its parameterisation can be found in the Supplement, and the Python (v3.8) code is freely available from github (https://github.com/lwyakob/COVIDsaturates).

SARS-CoV-2: severe acute respiratory syndrome coronavirus 2.

Model compartments are: ‘S’usceptible, ‘E’xposed, ‘I’nfectious, ‘P’re-critical infectious, ‘C’ritically ill, ‘D’ead and ‘R’ecovered. The three functional forms are shown for the force of infection, λ. Each is a product of the infectious proportion of the population and the transmission coefficient, β. The transmission coefficients are differentiated between the three functional forms using primes. The transmission coefficient is itself the product of the transmission probability per contact with an infectious individual and the contact rate. Under a linearly density-dependent assumption, the contact rate increases linearly with the total population, N. This numerator N cancels out the N denominator which is why β` is multiplied by the number of infectious individuals instead of the proportion. The saturating density-dependent formulation assumes the force of infection is a product of the transmission coefficient and a function that increases contact rate nonlinearly with population density as informed by parameter θ derived from analysing England’s regional mortality data.

### Ethical statement

No ethical clearance was needed for this publication because all data which were freely available from the Office for National Statistics were anonymised.

## Results

### Evidence for saturating density dependence in COVID-19-associated deaths

**Figure 2:**
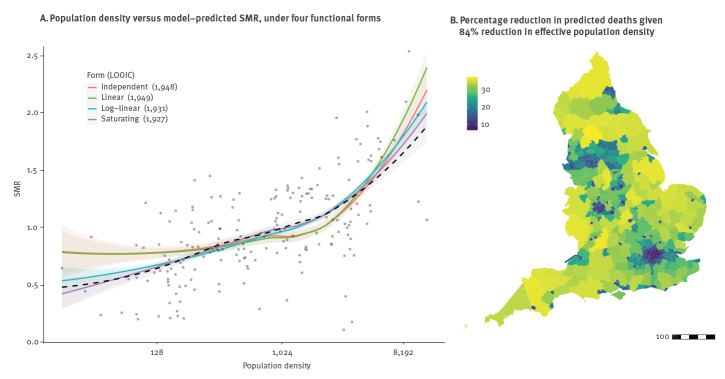
Dependence of observed vs age-specific expected mortality rates (standardised mortality ratio) on population density, England, March–July 2020

LOOIC: leave-one-out information criterion; SMR: standardised mortality ratio.

COVID-19-associated deaths appeared to be strongly correlated with population density, aligning with the rural/urban disparities demonstrated in Office for National Statistics bulletins (Supplementary Figure S2 shows deaths were higher for urban settings [21]). Adjusting for potential confounders (age distribution, deprivation, ethnic distribution, proportion of key workers within the local population) via a negative binomial generalised linear model, a saturating dependence on population density provided the best fit to total local authority mortality rates over the period 3 March to 31 July with respect to the LOOIC (Figure 2A). Models independent of, or linearly dependent on, density performed similarly since the fitted linear trend was negligibly small, and both performed worse than log-linear and saturating forms. The chosen model suggests a 3.6-fold (90% credible interval (CrI): 2.44–5.28) increase in mortality rate between the lowest and highest density areas, on the saturated scale. 

A. Four forms of density dependence (and loess curve, dashed black line) are illustrated in the left panel, with LOOIC values (in brackets after each model type) for each fit demonstrating superiority of the saturating density-dependent function. Population density has units of people per km^2^. For the saturating function, fitted θ = 0.05.

B. The heterogeneous impact of 84% effective density reduction on the proportional reduction in predicted mortality among the freely moving population according to the saturating model is mapped in the right panel.

Owing to the heightened risk of earlier outbreak seeding for higher density areas, we repeated the analysis, additionally adjusting for the lag of the local epidemic behind the national. The saturating model was retained as the best fit (Table) and suggested a similar increase in mortality rate of fourfold (90% CrI: 2.15–7.08).

**Table:** Model comparison for explaining variation in COVID-19 mortality rates, England, March–July 2020

Model form	LOOIC	LOOIC SE	Difference elpd	Difference elpd SE
Saturating	1,927	25.4	0.0	0.0
Log-linear	1,931	25.6	−1.7	1.4
Independent	1,948	23.4	−10.2	4.6
Linear	1,949	23.8	−10.7	4.3

elpd: expected log pointwise predictive density; COVID-19: coronavirus disease; LOOIC: leave one out information criterion; SE: standard error.

Models additionally account for the time the local epidemic lags behind the national and are compared on LOOIC, with all compared with the optimal model (saturating form) in the first row. Saturating and log-linear forms are not clearly distinguishable from each other, but both appear preferable over the independent and linear forms.

Under the saturating density-dependent model, the impact of lockdown on reducing transmission among mobile individuals, and consequently on deaths, is heterogeneous, having greatest benefit in regions with low population density (> 30% reduction in projected deaths for example in Devon, Herefordshire and the Derbyshire Dales) but reduced benefit in high-density regions (ca 5–7% reduction for the London boroughs of Tower Hamlets, Hackney, Islington and Camden) (Figure 2B). These results were retained when accounting for the lag of the local epidemic behind the national (Supplementary Figure S3).

### Projecting SARS-CoV-2 resurgence after lockdown is released

A full-text review of 100 epidemiological models of SARS-CoV-2 published until 19 June 2020 showed that 70% explicitly assumed that contact rate between people (and hence transmission) is unaffected by population density (see Supplement for details of the models). Of the remaining 30% of models, all assumed a linear relationship between population density and transmission.

**Figure 3:**
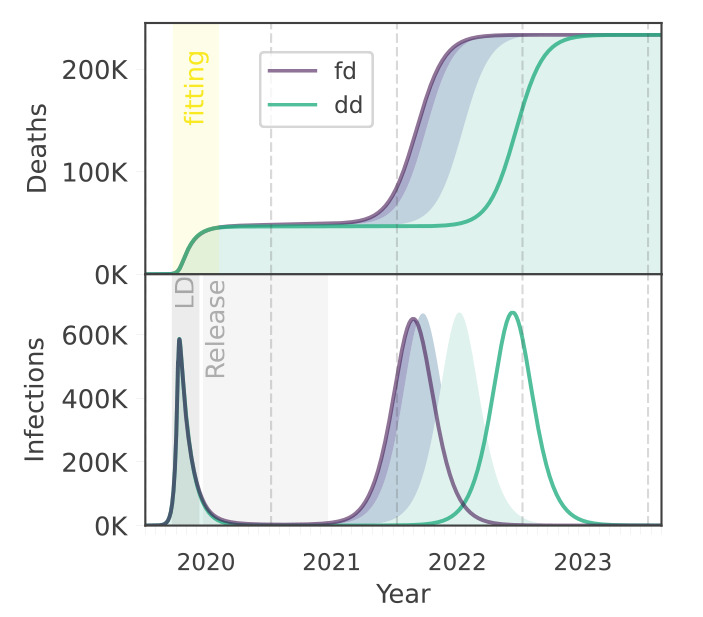
Population density and SARS-CoV-2-associated mortality and infection dynamics following the release of lockdown, by transmission assumptions, England, March–July 2020

COVID-19: coronavirus disease; dd: density-dependent; fd: frequency-dependent; LD: lockdown; K: ×1,000; SARS-CoV-2: severe acute respiratory syndrome coronavirus 2.

We used a metapopulation model to simulate the infection dynamics among freely moving as well as locked-down individuals, incorporating transmission terms that can accommodate density-independent (referred to as ‘frequency-dependent’) as well as linearly and saturating density-dependent assumptions. While all functional forms performed equivalently in fitting mortality data leading up to lockdown, dynamics under alternate assumptions may diverge markedly during and following the phase when lockdown is released (Figure 3). We note that we ignored any adaptive public health responses (i.e. additional interventions) curbing the second wave - this comparison was intended to illustrate the consequences to projected dynamics of alternative assumptions underlying density and transmission.

COVID-19 associated mortality (top) and infection dynamics (bottom) following the release of lockdown under the three different transmission terms (fd, linearly dd and saturating dd). Lighter filled areas illustrate saturating dd dynamics for lower population density (where England’s density is set to equal that of London at 5,700 people per km^2^, the average English population density at 430 people per km^2^ or Cornwall at 160 people per km^2^). These simulations show a 1-year release of locked-down individuals and infection preventative behaviours (e.g. face masks) that halve the transmission rate per contact (grey band denoted ‘Release’). Details of model-fitting are in the Supplement.

Although final epidemic size and total deaths were equivalent for the alternative classical assumptions (fd and linearly dd), transmission was delayed by almost a year under a density- vs frequency-dependent model (Figure 3). This delay occurs because only under the density-dependent assumption, the force of infection is reduced while any part of the population remains locked down. At the very high densities of London populations, locking down 84% of people under our saturating density-dependent model had an impact most similar to a frequency-dependent assumption. This means that, if the density of England’s entire population was equivalent to the density found in London, infection dynamics and deaths resulting from a saturating density-dependent model most closely match the frequency-dependent projections. However, London has a population density that is an order of magnitude higher than the next most populated region in England, and projected infection dynamics diverged more considerably under scenarios reflecting densities experienced outside of the capital. The force of infection and the timing of peak prevalence for the saturating density-dependent model is constrained between the frequency- and linearly density-dependent versions [20], with lower densities tending towards the latter.

**Figure 4:**
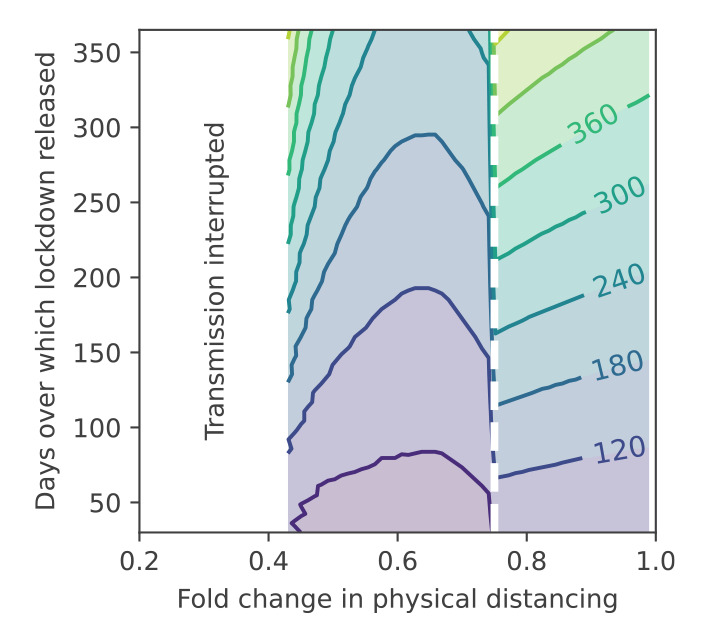
Consequences of density dependence on intensive care unit capacity, England, March–July 2020

Assuming a maximum national capacity of 5,000 intensive care unit (ICU) beds, we assessed the difference between these temporal limits in the projected duration between the release of lockdown and a second wave of infection exceeding ICU capacity (Figure 4). Threshold levels of physical distancing to interrupt transmission (i.e. maintain an effective reproduction number < 1) are similar for both classical models. However, where interventions failed to achieve this threshold, density-dependent transmission resulted in a delay of more than a year before the ICU capacity was exceeded. This is contingent on the timeframe across which lockdown is released, whereby more gradual releases extend delays.

Difference (in days, contours) between frequency- and density-dependent models in their projected time before ICU capacity is exceeded, as a function of lockdown release schedule and effectiveness of physical distancing. The dashed vertical line marks minimum physical distancing required to prevent exceeding ICU capacity in the frequency-dependent model.

## Discussion

Projections of COVID-19 infection dynamics following the release of a huge proportion of the population from lockdown comprise an urgent and critical component of public health decision-making [22]. The classical forms of modelling infectious diseases among populations have been used interchangeably by different research groups because, under most plausible circumstances, they exhibit equivalent dynamics. In March 2020, England locked down more than four-fifths of its population. For most, this fundamentally altered the rate at which people made contact. Under the circumstance of millions of people easing out of lockdown, substantial differences between projections from a frequency- and density-dependent transmission assumption emerge. Most notably, density dependence results in delayed infection resurgence and, contingent on the timeframe across which lockdown is released and the effectiveness of physical distancing, this delay can extend to more than a year.

The delay is a function of a fundamental aspect of density-dependent transmission: lower host densities reduce the force of infection, and there is a threshold host density below which an infection cannot spread. Despite its origins in human infectious disease modelling [23], the existence of this threshold has historically had limited epidemiological application. The phenomenon is discussed more widely in wildlife disease ecology [24] where it underlies key disease control decisions such as culling [25]. Current expectation is that lockdowns, either full or in a more moderate or localised form, will be reimplemented when cases start increasing again. Density effects and thresholds are particularly pertinent in the current COVID-19 pandemic during which extreme fluctuations in mobile human density are likely to continue.

Analysing COVID-19-associated deaths across different regions in England, and accounting for known major confounders [9], the nonlinear increase in deaths with population density was adequately captured by neither classical form of modelling transmission. Using a function that captures the saturating increase in deaths with population density resulted in an expedited resurgence compared with a linearly density-dependent model and a delayed resurgence compared with the popularly used frequency-dependent model.

Less populated areas were shown to have fewer deaths per capita (as per England’s mortality data) and slower resurgences following the release of lockdown. This provides more achievable targets and considerably more lead time for health services to prepare than would otherwise be anticipated. It also highlights a hazard. During and after releases from lockdown, in order to fit a prolonged lag in cases, transmission rates derived from most current (frequency-dependent) models will underestimate the effective reproduction number. This could exaggerate the perceived effectiveness of ongoing interventions, such as physical distancing or face masks, with potentially serious consequences.

Our study is limited by the fact that we do not have comprehensive data on how contact rates were affected before and over the lockdown period for individuals inhabiting regions of differing population density. We also do not know where people were infected, only where they were when they died. Instead we had to resort to mortality rates and locations as a proxy. It is possible, for example, that contact rates are not affected by dramatic shifts in population density regardless of baseline levels (i.e. the average England resident came into contact with as many individuals during lockdown as before lockdown, satisfying a frequency-dependent assumption), and that the increased per capita fatality seen in more densely populated regions has an alternative, thus far unidentified, explanation. Mobile phone applications developed to notify participants of urgent health information have already gained millions of users in the current coronavirus context [26]. Piggy-backing on these efforts could help substantiate the evidence for the contact-density relationships we have identified.

Owing to the highly complex interactions between population characteristics, behaviours and mortality risk, the association discovered between saturating density and mortality rates may remain confounded by factors not considered here. Moreover, the criterion used for model comparison depends on an independence assumption which may not hold between neighbouring LTLA. Work is ongoing to characterise the patterns of spatial correlation in mortality at the LTLA level.

## Conclusion

Infectious diseases are emerging at an unprecedented rate and the upwards trend in global travel and urbanisation increases the likelihood of pandemics. Their success in controlling SARS-CoV-2 means that widescale lockdowns will not only continue to be enforced as this pandemic progresses, but they are likely to be more readily applied in future emergencies. It is crucial that we use the current opportunity to collect data to inform more precise forms of how contact rates are altered at varying stages of lockdown. Future work should also address whether the feature of saturating density dependence we have identified from England’s data are generalisable to other countries. Incorporating realistic contact-density relationships into the transmission term of population-level mathematical models will improve precision of their projections and their utility in public health decision-making.

## Supplementary Data

373000 Supplement
