# Supplementary material for: The importance of saturating density dependence for population-level predictions of SARS-CoV-2 resurgence compared with density-independent or linearly density-dependent models, England, 23 March to 31 July 2020
Source: Euro Surveill. 2021 Dec 9;26(49):2001809. doi: 10.2807/1560-7917.ES.2021.26.49.2001809 (PMC8662798; doi:10.2807/1560-7917.ES.2021.26.49.2001809)
Supplement: Supplement [file 20-01809_YAKOB_Supplement.pdf]

Supplementary Materials

This supplementary material is hosted by Eurosurveillance as supporting information alongside the article 'The importance of saturating density dependence for population-level predictions of SARS-CoV-2 resurgence: comparison of models accommodating this to density-independent or linearly density-dependent models using data from England, 23 March to 31 July 2020', on behalf of the authors, who remain responsible for the accuracy and appropriateness of the content. The same standards for ethics, copyright, attributions and permissions as for the article apply. Supplements are not edited by Eurosurveillance and the journal is not responsible for the maintenance of any links or email addresses provided therein.

Supplementary Materials and Methods

Google Scholar search by year '2020' on June 19th 2020:

coronavirus, OR covid19, OR covid-19 "'(mathematical OR simulation OR transmission) model'"

613 results were returned and ordered by relevance. These were consecutively reviewed to ascertain if they used SIR-related models and whether they declared (either explicitly stating or in presented equations) which functional form of transmission was used in their analysis, until 100 relevant articles were found for inclusion in this review. The following are the list of papers sorted by whether they use linearly density- or frequency-dependent transmission.

Linearly density-dependent transmission

1. Aleta, A., et al., Modeling the impact of social distancing, testing, contact tracing and household quarantine on second-wave scenarios of the COVID-19 epidemic. medRxiv, 2020.

2. Arenas, A., et al., A mathematical model for the spatiotemporal epidemic spreading of COVID19. MedRxiv, 2020.

3. Bentout, S., A. Chekroun, and T. Kuniya, Parameter estimation and prediction for coronavirus disease outbreak 2019 (COVID-19) in Algeria. AIMS Public Health, 2020. 7(2): p. 306.

4. Boudrioua, M.S. and A. Boudrioua, Predicting the COVID-19 epidemic in Algeria using the SIR model. medRxiv, 2020.

5. Chatterjee, K., et al., Healthcare impact of COVID-19 epidemic in India: A stochastic mathematical model. Medical Journal Armed Forces India, 2020. 76(2): p. 147-55.

6. Diop, B.Z., et al., The relatively young and rural population may limit the spread and severity of Covid-19 in Africa: a modelling study. BMJ global health, 2020. 5(5): p. e002699.

7. Eguiluz, V.M., et al., Risk of secondary infection waves of COVID-19 in an insular region: the case of the Balearic Islands, Spain. medRxiv, 2020.

8. Engbert, R., et al., Sequential data assimilation of the stochastic SEIR epidemic model for regional COVID-19 dynamics. medRxiv, 2020.

9. Giordano, G., et al., A SIDARTHE model of COVID-19 epidemic in Italy. arXiv, 2020.

10. González, R.E., C. Estupiñán-López, and C.A.C. Morales, An adapted ODE model to study the Dynamics of SARS-Cov-2 Infection (COVID-19): different scenarios for Brazil and other countries. Research Square, 2020.

11. Guinet, A., A modelling of COVID-19 outbreak with a linear compartmental model. HAL, 2020.

12. Kissler, S.M., et al., Projecting the transmission dynamics of SARS-CoV-2 through the postpandemic period. Science, 2020. 368(6493): p. 860-868.

13. Mbabazi, F.K., et al., A Mathematical Model Approach for Prevention and Intervention Measures of the COVID–19 Pandemic in Uganda. MedRxiv, 2020.

14. Ng, K.Y. and M.M. Gui, COVID-19: Development of a robust mathematical model and simulation package with consideration for ageing population and time delay for control action and resusceptibility. Physica D: Nonlinear Phenomena, 2020: p. 132599.

15. Pai, C., A. Bhaskar, and V. Rawoot, Investigating the dynamics of COVID-19 pandemic in India under lockdown. Chaos, Solitons & Fractals, 2020. 138: p. 109988.

16. Pais, R.J. and N. Taveira, Predicting the evolution and control of the COVID-19 pandemic in Portugal. F1000Research, 2020. 9(283): p. 283.

17. Pedro, S.A., et al., Conditions for a second wave of COVID-19 due to interactions between disease dynamics and social processes. medRxiv, 2020.

18. Peirlinck, M., et al., Outbreak dynamics of COVID-19 in China and the United States. Biomechanics and modeling in mechanobiology, 2020: p. 1-15.

19. Pellis, L., et al., Challenges in control of Covid-19: short doubling time and long delay to effect of interventions. arXiv, 2020.

20. Prem, K., et al., The effect of control strategies to reduce social mixing on outcomes of the COVID-19 epidemic in Wuhan, China: a modelling study. The Lancet Public Health, 2020. 5(5): p. E261-E270.

21. Prieto, F., et al., COVID-19 Impact Estimation on ICU Capacity at Andalusia, Spain, Using Artificial Intelligence. Research Square, 2020.

22. Rasha, M. and S. Balamuralitharan, A study on COVID-19 transmission dynamics: Stability analysis of SEIR model with Hopf bifurcation for effect of time delay. Research Square, 2020.

23. Saha, S. and S. Saha, The impact of the undetected COVID-19 cases on its transmission dynamics. medRxiv, 2020.

24. Sarma, U. and B. Ghosh, Quantitative modeling and analysis show country-specific optimization of quarantine measures can potentially circumvent COVID19 infection spread post lockdown. medRxiv, 2020.

25. Schwartz, I.B., et al., Predicting the impact of asymptomatic transmission, non-pharmaceutical intervention and testing on the spread of COVID19 COVID19. medRxiv, 2020.

26. Wahid, A., et al., The Epidemiology of COVID-19 and applying Non Pharmaceutical interventions by using the Susceptible, Infectious Recovered epidemiological Model in Pakistan. medRxiv, 2020.

27. Wickramaarachchi, T. and S. Perera, Optimal Control Measures to Combat COVID 19 Spread in Sri Lanka: A Mathematical Model Considering the Heterogeneity of Cases. medRxiv, 2020.

28. Wickramaarachchi, T., S. Perera, and S. Jayasinghe, COVID-19 epidemic in Sri Lanka: A mathematical and computational modelling approach to control. medRxiv, 2020.

29. Yafia, R., Modeling and Dynamics in Epidemiology, COVID19 with Lockdown and Isolation Effect: Application to Moroccan Case. medRxiv, 2020.

30. Zongo, P., et al., A model of covid-19 transmission to understand the effectiveness of the containment measures: application to French data. HAL, 2020.

Frequency-dependent transmission

1. Abrigo, M.R., et al., Projected Disease Transmission, Health System Requirements, and Macroeconomic Impacts of the Coronavirus Disease 2019 (COVID-19) in the Philippines. 2020, Discussion Paper Series.

2. Al-Shammari, A.A., et al., Real-time tracking and forecasting of the COVID-19 outbreak in Kuwait: a mathematical modeling study. medRxiv, 2020.

3. Ali, M., M. Imran, and A. Khan, Can medication mitigate the need for a strict lock down?: A mathematical study of control strategies for COVID-19 infection. medRxiv, 2020.

4. Ali, M., et al., The role of asymptomatic class, quarantine and isolation in the transmission of COVID-19. Journal of Biological Dynamics, 2020. 14(1): p. 389-408.

5. Alshammari, F.S., A mathematical model to investigate the transmission of COVID-19 in the Kingdom of Saudi Arabia. medRxiv, 2020.

6. Armstrong, E., M. Runge, and J. Gerardin, Identifying the measurements required to estimate rates of COVID-19 transmission, infection, and detection, using variational data assimilation. MedRXiv, 2020.

7. Assob, J.-C., D. Dongo, and P.E. Nguimkeu, Early Dynamics of Transmission and Projections of COVID-19 in Some West African Countries. 2020.

8. Atkeson, A., What will be the economic impact of COVID-19 in the US? Rough estimates of disease scenarios. 2020, National Bureau of Economic Research.

9. Ayoub, H.H., et al., Characterizing key attributes of the epidemiology of COVID-19 in China: Model-based estimations. medRxiv, 2020.

10. Bherwani, H., et al., Exploring Dependence of COVID-19 on Environmental Factors and Spread Prediction in India. 2020.

11. Blasius, B., Power-law distribution in the number of confirmed COVID-19 cases. arXiv, 2020.

12. Brugnago, E.L., et al., How relevant is the decision of containment measures against COVID-19 applied ahead of time? arXiv, 2020.

13. Casella, F., Can the COVID-19 epidemic be managed on the basis of daily data? arXiv, 2020.

14. Chen, X. and Z. Qiu, Scenario analysis of non-pharmaceutical interventions on global COVID-19 transmissions. arXiv, 2020.

15. Chudik, A., M.H. Pesaran, and A. Rebucci, Voluntary and mandatory social distancing: Evidence on covid-19 exposure rates from chinese provinces and selected countries. 2020, National Bureau of Economic Research.

16. Cuevas, J.A.G., SEI1I2HRSVM model applied to the coronavirus pandemic (COVID-19) in Paraguay. arXiv, 2020.

17. Davies, N.G., et al., Age-dependent effects in the transmission and control of COVID-19 epidemics. Nature Medicine, 2020.

18. Davies, N.G., et al., Effects of non-pharmaceutical interventions on COVID-19 cases, deaths, and demand for hospital services in the UK: a modelling study. The Lancet Public Health, 2020.

19. Davies, N.G., et al., The impact of Coronavirus disease 2019 (COVID-19) on health systems and household resources in Africa and South Asia. medRxiv, 2020.

20. Dehning, J., et al., Inferring change points in the spread of COVID-19 reveals the effectiveness of interventions. Science, 2020.

21. Duczmal, L.H., et al., Vertical social distancing policy (‘Isolamento Vertical’) is ineffective to contain the coronavirus COVID-19 pandemic. Cadernos de Saúde Pública, 2020.

22. Dziugys, A., et al., Simplified model of Covid-19 epidemic prognosis under quarantine and estimation of quarantine effectiveness. medRxiv, 2020.

23. Eikenberry, S.E., et al., To mask or not to mask: Modeling the potential for face mask use by the general public to curtail the COVID-19 pandemic. Infectious Disease Modelling, 2020. 5: p. 293-308.

24. Francis, A., et al., Projected ICU and Mortuary load due to COVID-19 in Sydney. medRxiv, 2020.

25. Gatto, M., et al., Spread and dynamics of the COVID-19 epidemic in Italy: Effects of emergency containment measures. Proceedings of the National Academy of Sciences, 2020. 117(19): p. 10484-10491.

26. Gupta, M., et al., Transmission dynamics of the COVID-19 epidemic in India and modelling optimal lockdown exit strategies. medRxiv, 2020.

27. Harris, J.E., The Coronavirus Epidemic Curve is Already Flattening in New York City. 2020, National Bureau of Economic Research.

28. Hasan, A., et al., A new estimation method for COVID-19 time-varying reproduction number using active cases. arXiv, 2020.

29. Hochberg, M.E., Importance of suppression and mitigation measures in managing COVID-19 outbreaks. arXiv, 2020.

30. Iboi, E.A., et al., Mathematical Modeling and Analysis of COVID-19 pandemic in Nigeria. medRxiv, 2020.

31. Ivorra, B., et al., Mathematical modeling of the spread of the coronavirus disease 2019 (COVID-19) taking into account the undetected infections. The case of China. Communications in nonlinear science and numerical simulation, 2020. 88: p. 105303.

32. Jitsuk, N.C., et al., Effect of the Songkran festival on COVID-19 transmission in Thailand. Asian Pacific Journal of Tropical Medicine, 2020. 13(7): p. 331-332.

33. Kassa, S.M., J.B. Njagarah, and Y.A. Terefe, Analysis of the mitigation strategies for COVID-19: from mathematical modelling perspective. Chaos, Solitons & Fractals, 2020. 138: p. 109968.

34. Ke, R., et al., Fast spread of COVID-19 in Europe and the US suggests the necessity of early, strong and comprehensive interventions. medRxiv, 2020.

35. Kennedy, D.M., et al., Modeling the effects of intervention strategies on COVID-19 transmission dynamics. Journal of Clinical Virology, 2020. 128: p. 104440.

36. Khailaie, S., et al., Estimate of the development of the epidemic reproduction number Rt from Coronavirus SARS-CoV-2 case data and implications for political measures based on prognostics. medRxiv, 2020.

37. Khajanchi, S., et al., Dynamics of the COVID-19 pandemic in India. arXiv, 2020.

38. Kouakep, Y., et al., Modelling the anti-COVID19 individual or collective containment strategies in Cameroon, in Epi-Ndere. 2020: At University of Ngaoundere (Cameroon).

39. Kumar, S., S. Sharma, and N. Kumari, Future of COVID-19 in Italy: A mathematical perspective. arXiv, 2020.

40. Kuzdeuov, A., et al., A Network-Based Stochastic Epidemic Simulator: Controlling COVID-19 with Region-Specific Policies. medRxiv, 2020.

41. Kyagulanyi, A., et al., Risk analysis and prediction for COVID19 demographics in low resource settings using a python desktop app and excel models. medRxiv, 2020.

42. Leng, T., et al., The effectiveness of social bubbles as part of a Covid-19 lockdown exit strategy, a modelling study. medRxiv, 2020.

43. López, L. and X. Rodo, A modified SEIR model to predict the COVID-19 outbreak in Spain and Italy: simulating control scenarios and multi-scale epidemics. Available at SSRN 3576802, 2020.

44. Luo, J., Predictive Monitoring of COVID-19. SUTD Data-Driven Innovation Lab, 2020.

45. Lyra, W., et al., COVID-19 pandemics modeling with SEIR (+ CAQH), social distancing, and age stratification. The effect of vertical confinement and release in Brazil. medRxiv, 2020.

46. MANOU-ABI, S. and J. BALICCHI, Analysis of the COVID-19 epidemic in french overseas department Mayotte based on a modified deterministic and stochastic SEIR model. medRxiv, 2020.

47. Mukaddes, A.M.M. and M. Sannyal, Transmission Dynamics of COVID-19 in Bangladesh-A Compartmental Modeling Approach. Research Square, 2020.

48. Neto, O.P., et al., COVID-19 mathematical model reopening scenarios for Sao Paulo-Brazil. medRxiv, 2020.

49. Nyabadza, F., et al., Modelling the potential impact of social distancing on the COVID-19 epidemic in South Africa. medRxiv, 2020.

50. Ogbunugafor, C.B., et al., Variation in SARS-CoV-2 free-living survival and environmental transmission can modulate the intensity of COVID-19 outbreaks. medRxiv, 2020.

51. Pant, R., et al., COVID-19 Epidemic Dynamics and Population Projections from Early Days of Case Reporting in a 40 million population from Southern India. medRxiv, 2020.

52. Picchiotti, N., et al., COVID-19 Italian and Europe epidemic evolution: A SEIR model with lockdown-dependent transmission rate based on Chinese data. Available at SSRN 3562452, 2020.

53. Picchiotti, N., et al., COVID-19 pandemic: a mobility-dependent SEIR model with undetected cases in Italy, Europe and US. arXiv, 2020.

54. Prabhakaran, H., Spread of the Novel Coronavirus (SARS-CoV-2): Modeling and Simulation of Control Strategies. medRxiv, 2020.

55. Rahman, M.M., et al., Impact of control strategies on COVID-19 pandemic and the SIR model based forecasting in Bangladesh. medRxiv, 2020.

56. Rajesh, A., et al., CoVID-19 prediction for India from the existing data and SIR (D) model study. medRxiv, 2020.

57. Rapolu, T., et al., A Time-Dependent SEIRD Model for Forecasting the COVID-19 Transmission Dynamics. medRxiv, 2020.

58. Rawson, T., et al., How and When to End the COVID-19 Lockdown: An Optimization Approach. Frontiers in Public Health, 2020. 8: p. 262.

59. Reis, R.F., et al., Characterization of the COVID-19 pandemic and the impact of uncertainties, mitigation strategies, and underreporting of cases in South Korea, Italy, and Brazil. Chaos, Solitons & Fractals, 2020: p. 109888.

60. Rouabah, M.T., A. Tounsi, and N.-E. Belaloui, Early dynamics of COVID-19 in Algeria: a model-based study. arXiv, 2020.

61. Salomon, J.A., Defining high-value information for COVID-19 decision-making. medRxiv, 2020.

62. Sardar, T., S.S. Nadim, and J. Chattopadhyay, Assessment of 21 days lockdown effect in some states and overall India: a predictive mathematical study on COVID-19 outbreak. arXiv, 2020.

63. Tuite, A.R., D.N. Fisman, and A.L. Greer, Mathematical modelling of COVID-19 transmission and mitigation strategies in the population of Ontario, Canada. CMAJ, 2020. 192(19): p. E497-E505.

64. Tuite, A.R., et al., Risk for COVID-19 Resurgence Related to Duration and Effectiveness of Physical Distancing in Ontario, Canada. Annals of Internal Medicine, 2020(M20-2945).

65. Verachi, F., L.G. Trussoni, and L. Lanzi, CoViD-19 in Italy: a mathematical model to analyze the epidemic containment strategy and the economic impacts. medRxiv, 2020.

66. Xu, R., et al., Weather Conditions and COVID-19 Transmission: Estimates and Projections. Available at SSRN 3593879, 2020.

67. Yang, P., et al., The effect of multiple interventions to balance healthcare demand for controlling COVID-19 outbreaks: a modelling study. medRxiv, 2020.

68. Zhang, J., et al., Changes in contact patterns shape the dynamics of the COVID-19 outbreak in China. Science, 2020.

69. Zhang, Y., et al., Applicability of time fractional derivative models for simulating the dynamics and mitigation scenarios of COVID-19. Chaos, Solitons & Fractals, 2020: p. 109959.

70. Matrajt, L. and T. Leung, Early Release-Evaluating the Effectiveness of Social Distancing Interventions to Delay or Flatten the Epidemic Curve of Coronavirus Disease. Emerging Infectious Diseases, 2020. 26(8).

Mathematical model equations

Free-moving subpopulation:

$$S_t = S_{t-1} - S_{t-1}(\sigma\lambda + \varepsilon)(1-\phi) - (\phi S_{t-1}) + \left(\psi S^2_{L,t-1}\right) + \left(\kappa S_{L,t-1}\right)$$

$$E_t = E_{t-1} + [\sigma\lambda S_{t-1} - (\alpha + \varepsilon)E_{t-1}](1-\phi) - (\phi E_{t-1}) + \left(\psi E^2_{L,t-1}\right) + \left(\kappa E_{L,t-1}\right)$$

$$I_t = I_{t-1} + [(1-\rho)\alpha E_{t-1} - (\gamma + \varepsilon)I_{t-1}](1-\phi) - (\phi I_{t-1}) + \left(\psi I^2_{L,t-1}\right) + \left(\kappa I_{L,t-1}\right)$$

$$P_t = P_{t-1} + [\rho\alpha E_{t-1} - (\nu + \varepsilon)P_{t-1}](1-\phi) - (\phi P_{t-1}) + \left(\psi P^2_{L,t-1}\right) + \left(\kappa P_{L,t-1}\right)$$

$$C_t = C_{t-1} + [\nu P_{t-1} - (\mu\tau + \varepsilon + (1-\mu)\omega)C_{t-1}](1-\phi) - (\phi C_{t-1}) + \left(\psi C^2_{L,t-1}\right) + \left(\kappa C_{L,t-1}\right)$$

$$R_t = R_{t-1} + [\gamma I_{t-1} + (1-\mu)\omega C_{t-1} - \varepsilon R_{t-1}](1-\phi) - (\phi R_{t-1}) + \left(\psi R^2_{L,t-1}\right) + \left(\kappa R_{L,t-1}\right)$$

$$D_t = D_{t-1} + \mu\tau C_{t-1}$$

Locked down subpopulation:

$$S_{L,t} = S_{L,t-1} - S_{L,t-1}(\lambda + \kappa) + (\phi S_{t-1}) - (\psi S^2_{t-1}) + (1-\phi)\varepsilon S_{t-1}$$

$$E_{L,t} = E_{L,t-1} + \lambda S_{L,t-1} - (\alpha + \kappa)E_{L,t-1} + (\phi E_{t-1}) - \left(\psi E^2_{L,t-1}\right) + (1-\phi)\varepsilon E_{L,t-1}$$

$$I_{L,t} = I_{L,t-1} + (1-\rho)\alpha E_{L,t-1} - (\gamma + \kappa)I_{L,t-1} + (\phi I_{t-1}) - \left(\psi I^2_{L,t-1}\right) + (1-\phi)\varepsilon I_{L,t-1}$$

$$P_{L,t} = P_{L,t-1} + \rho\alpha E_{L,t-1} - (\nu + \kappa)P_{L,t-1} + (\phi P_{t-1}) - \left(\psi P^2_{L,t-1}\right) + (1-\phi)\varepsilon P_{L,t-1}$$

$$C_{L,t} = C_{L,t-1} + \nu P_{L,t-1} - (\mu\tau + \kappa + (1-\mu)\omega)C_{L,t-1} + (\phi C_{t-1}) - \left(\psi C^2_{L,t-1}\right) + (1-\phi)\varepsilon C_{L,t-1}$$

$$R_{L,t} = R_{L,t-1} + \gamma I_{L,t-1} + (1-\mu)\omega C_{L,t-1} - \kappa R_{L,t-1} + (\phi R_{t-1}) - \left(\psi R^2_{L,t-1}\right) + (1-\phi)\varepsilon R_{L,t-1}$$

$$D_{L,t} = D_{L,t-1} + \mu\tau C_{L,t-1}$$

The transmission rate in the absence of any intervention was set assuming an R_0 of 3 (*S1*). Data captured deaths pre-lockdown, during lockdown and just following the initial release of individuals after lockdown. During lockdown, the population split into two sub-populations. The transmission rates were generated by fitting deaths in the model to England's mortality data. At the same time as lockdown, physical distancing also reduced per capita contact rates among the free-moving population:

$$\sigma = \begin{cases} range(0.1 - 1) & if\ t > lockdown \\ 1 & otherwise \end{cases}$$

For the full range of physical distancing, the model was refitted to the mortality data (more stringent physical distancing among free-movers requiring less of a reduction in the lockdown transmission rate, β_L). Least squares fitting using the Levenberg-Marquardt minimization algorithm was conducted using 'lmfit' in Python v3.8.

Supplementary figures

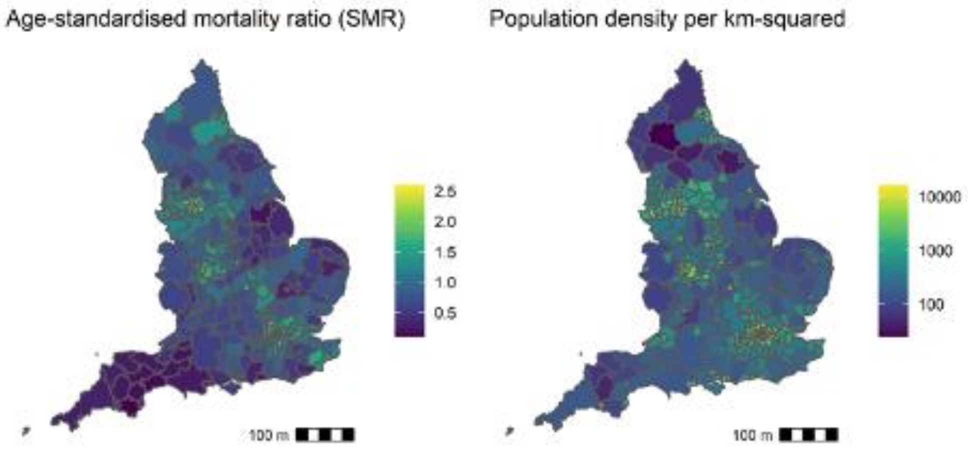

Fig S1. COVID-19 associated mortality and population density in England. Age-standardized mortality ratios are mapped in the left panel and population density per km^2 in the right, by lower-tier local authority.

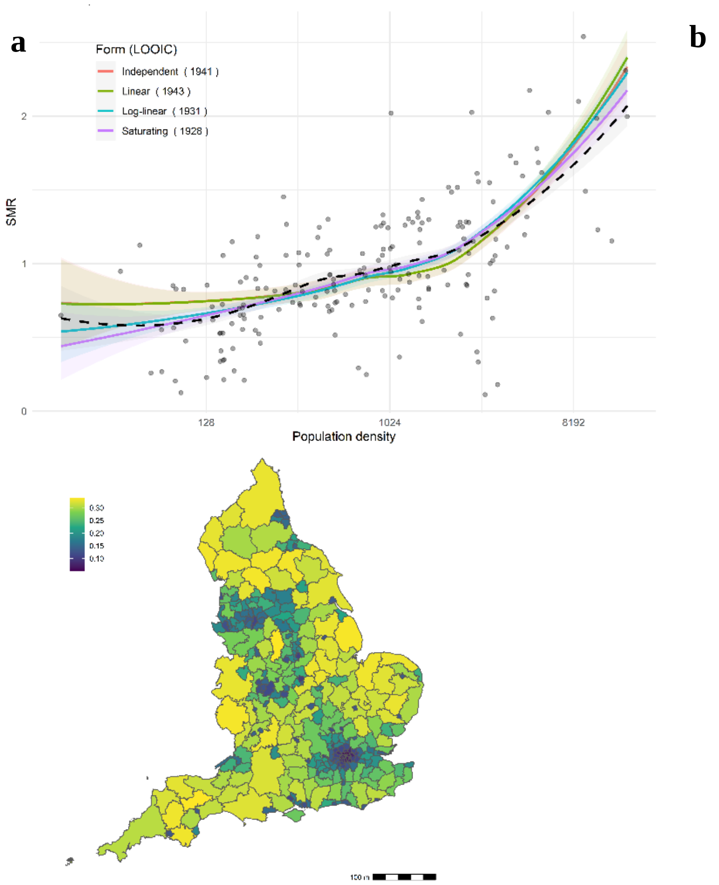

Fig S2. Dependence of observed versus age-specific expected mortality rates (standardized mortality ratio, SMR) on population density. The effects of different outbreak seed timings for different LTLAs are incorporated. **a)** Four forms of density dependence (and loess curve, dashed black line) are illustrated in the left panel, with LOOIC values for each fit demonstrating superiority of the saturating density-dependent function. **b)** The heterogeneous impact of 84% effective density reduction on the proportional reduction in predicted mortality among the freely moving population according to the saturating model is mapped in the right panel.

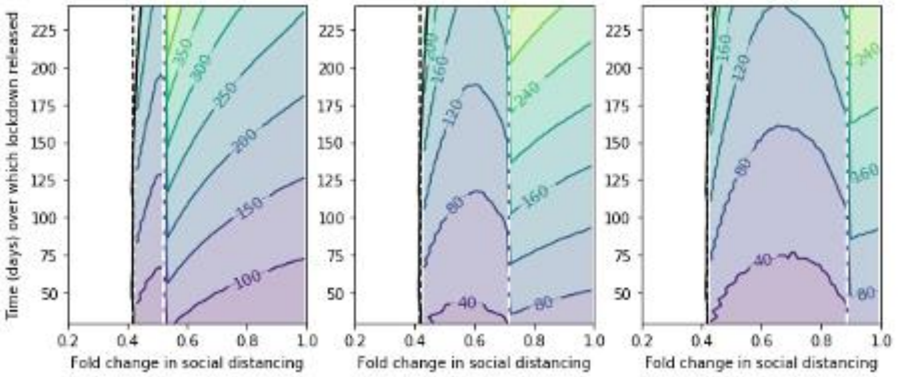

Fig. S3. Sensitivity analysis of movements between sub-populations. The difference between functional forms in projected time (contours=days) until the ICU capacity is exceeded by critically ill patients. Left: 50% movement (‘ε’), Middle: 100% ε, Right: 150% ε. For each value of ε the model was refitted to the data (the density-dependent model was insensitive to movement; for the frequency-dependent model, higher rates of movement required slightly less of a reduction in transmission among the lockdown sub-population). Black lines mark thresholds for interrupting transmission for density-independent (dashed) and -dependent (solid) models. Dashed white lines mark minimum physical distancing required to prevent immediate ICU inundation under the frequency-dependent model.

Supplementary tables

Table S1. Mathematical model variables

Variable	Definition
S_t , $S_{L,t}$	Susceptible population at time ‘t’; subscript ‘L’ denotes lockdown sub-population
E_t , $E_{L,t}$	Exposed population at time ‘t’
I_t , $I_{L,t}$	Infected population at time ‘t’
P_t , $P_{L,t}$	Pre-critically infected population at time ‘t’
C_t , $C_{L,t}$	Critically infected population at time ‘t’
R_t , $R_{L,t}$	Recovered population at time ‘t’
D_t , $D_{L,t}$	Dead population at time ‘t’

Table S2. Mathematical model parameters

Parameter	Definition	Value	Source
λ	Force of infection (composite of other parameters and variables)	n/a	n/a
β''	Transmission rate (saturating density dependence)		derived
β'	Transmission rate (linear density dependence)		derived
β	Transmission rate (frequency dependence)		derived
β_L	Transmission rate while under lockdown (frequency dependent)		derived
ε	Daily rate of movement from free-moving to lockdown sub-pop	0.1 but see sensitivity analysis section	
κ	Daily rate of movement from lockdown to free-moving sub-pop		
ϕ	Pulsed, mass movement of 84% free-movers into lockdown	0.84	*(S2)*
ψ	Daily rate of release of lockdown sub-pop	Wide range tested	
σ	Fold change in physical contacts among free-movers	Wide range tested	
α	Inverse of infection latent period	1/5.8	*(S3)*
ρ	Proportion of infected individuals becoming critically infected	0.02	*(S4)*
γ	Inverse of recovery period	1/5	*(S5)*
ν	Inverse of additional delay before symptoms become critical	1/7	*(S6)*
μ	Proportion of critically infected that die	0.427	*(S7)*
τ	Inverse of time for critically ill to die	1/7	*(S8)*
ω	Inverse of time for critically ill to recover	1/7.2	*(S7)*

Supplementary references

S1. Y. Liu, A. A. Gayle, A. Wilder-Smith, J. Rocklöv, The reproductive number of COVID-19 is higher compared to SARS coronavirus. J Travel Med 27, (2020).

S2. The Editors, The plight of essential workers during the COVID-19 pandemic. *The Lancet* **395**, 1587 (2020).

S3. J. A. Backer, D. Klinkenberg, J. Wallinga, Incubation period of 2019 novel coronavirus (2019-nCoV) infections among travellers from Wuhan, China, 20–28 January 2020. *Eurosurveillance* **25**, 2000062 (2020).

S4. Z. Wu, J. M. McGoogan, Characteristics of and Important Lessons From the Coronavirus Disease 2019 (COVID-19) Outbreak in China: Summary of a Report of 72 314 Cases From the Chinese Center for Disease Control and Prevention. *Journal of the American Medical Association*, (2020).

S5. Y. Liu *et al.*, Viral dynamics in mild and severe cases of COVID-19. *Lancet Infectious Diseases* **20**, 656-657 (2020).

S6. W. Wang, J. Tang, F. Wei, Updated understanding of the outbreak of 2019 novel coronavirus (2019-nCoV) in Wuhan, China. *Journal of Medical Virology* **92**, 441-447 (2020).

S7. Public Health Scotland, "COVID-19 Statistical Report," (2020).

S8. X. Yang *et al.*, Clinical course and outcomes of critically ill patients with SARS-CoV-2 pneumonia in Wuhan, China: a single-centered, retrospective, observational study. *The Lancet Respiratory Medicine* **8**, 475-481 (2020).
